# Methylation of *RUNX3* Promoter 2 in the Whole Blood of Children with Ulcerative Colitis

**DOI:** 10.3390/genes13091568

**Published:** 2022-09-01

**Authors:** Emilia Dybska, Jan Krzysztof Nowak, Aleksandra Banaszkiewicz, Anna Szaflarska-Popławska, Jarosław Kierkuś, Jarosław Kwiecień, Urszula Grzybowska-Chlebowczyk, Jarosław Walkowiak

**Affiliations:** 1 Department of Pediatric Gastroenterology and Metabolic Diseases, Poznan University of Medical Sciences, 60-572 Poznan, Poland; 2 Department of Pediatric Gastroenterology and Nutrition, Medical University of Warsaw, 02-091 Warsaw, Poland; 3 Department of Pediatric Endoscopy and Gastrointestinal Function Testing, Collegium Medicum in Bydgoszcz, Nicolaus Copernicus University in Torun, 85-094 Bydgoszcz, Poland; 4 Department of Gastroenterology, Hepatology, Feeding Disorders and Pediatrics, The Children’s Memorial Health Institute, 04-730 Warsaw, Poland; 5 Department of Pediatrics, Faculty of Medical Sciences in Zabrze, Medical University of Silesia in Katowice, 41-800 Zabrze, Poland; 6 Department of Pediatrics, Faculty of Medical Sciences, Medical University of Silesia in Katowice, 40-752 Katowice, Poland

**Keywords:** *RUNX3*, DNA methylation, methylation-sensitive restriction enzyme, ulcerative colitis

## Abstract

Ulcerative colitis (UC) results from a complex interplay between the environment, gut microbiota, host genetics, and immunity. Runt-related transcription factor 3 (RUNX3) regulates Th1/Th2 balance and, thus, the synthesis of cytokines and inflammation. We aimed to analyze the dependence of *RUNX3* promoter 2 (*P2*) methylation level on: age, sex, body mass index (BMI), C-reactive protein (CRP), serum albumin, disease duration, Pediatric Ulcerative Colitis Activity Index (PUCAI), the Paris classification, and exposure to medications. This multicenter, cross-sectional study recruited hospitalized children with UC. Methylation of *RUNX3 P2* was measured with methylation-sensitive restriction enzymes in the whole blood DNA. Sixty-four children were enrolled, with a mean age of 14.5 ± 2.8 years. Half of them were female (51.6%), and the average BMI Z-score was −0.44 ± 1.14. The mean methylation of *RUNX3 P2* was 54.1 ± 13.3%. The methylation level of *RUNX3 P2* did not correlate with age, sex, nutritional status, CRP, albumin, PUCAI, or the extent of colitis (Paris E1–E4). *RUNX3 P2* methylation did not differ between patients recruited within two and a half months of diagnosis and children who had UC for at least a year. Current or past exposure to biologics, immunosuppressants, or steroids was not associated with *RUNX3 P2* methylation. Methylation of *RUNX3* promoter 2 in whole blood DNA does not seem to be associated with the characteristics of UC in children.

## 1. Introduction

Ulcerative colitis (UC) is a chronic inflammatory disease of the colon [1,2]. Damage to the intestinal barrier is driven by numerous infiltrating immune cells and alerted cytokine networks, with the important involvement of both innate and adaptive immune responses [3,4]. The inflamed gut mucosa hosts plenty of activated lymphocytes (T helper 2, T helper 1, regulatory T subsets), antigen-presenting cells (dendritic cells and macrophages), and natural killers [4,5]. Despite the elevated pool of T lymphocytes in the mucosa of patients with inflammatory bowel disease (IBD), the active disease associates with diminished anti-inflammatory forkhead box P3 CD4-positive regulatory T cells (FOXP3+CD4+ Tregs) in the peripheral blood [6]. Insufficient suppression of inflammation in UC is characterized by the synthesis of Th2-type cytokines and interferon-gamma (IFN-γ). These further aggravate the imbalance between pro- (IL-1, 6, 9, 13, 33, and TNF-α) and anti-inflammatory (IL-10, 37, and TGF-β) cytokines [3,4]. 

Alteration of cytokine gene expression by T cells is proposed to be subject to feed-forward regulation by the *RUNX3* gene [7], identified as an epistatic risk factor of UC [8]. The conserved runt domain-containing family consists of three genes (*RUNX1*, *RUNX2*, and *RUNX3*), among which *RUNX3* is both the simplest structurally and the smallest in size [9,10]. Two promoters—namely, CpG-poor distal P1 at 3′ and proximal P2 mapped within a conserved CpG island at 5′—transcriptionally regulate all *RUNX* family members [9,10,11]. Their dysregulation has been linked to both hematopoietic and gastrointestinal pathologies, including leukemia, spontaneous colitis, and gastric carcinogenesis [11,12]. Predominant expression of *RUNX3* in hematopoietic lineages orchestrates T-cell differentiation, dendritic cell maturation, and NK activation [10]. While genome-wide methylation profiling studies suggest *RUNX3* methylation variance in IBD [13,14], mouse models of cytokine gene expression have shown T-bet-dependent induction of Runx3 in CD4+ T lymphocytes. Both transcription factors bind to the *Ifng* promoter and the *Il4* silencer, providing an optimal synthesis of IFN-γ and reduction of the IL-4 pool. As IL-4 promotes Th2 differentiation, co-expression of Runx3 and T-bet regulates Th1/Th2 balance [7]. Given the known functional antagonism between Runx3 and zinc finger and BTB domain containing 7B (ThPOK), Runx3 directs CD4 to CD8 lineage switch or their transformation into Th17 cells. CD4+ T lymphocytes acquire Runx3 via the TGF-β/RORγt-mediated signaling pathway [5]. Moreover, ectopic expression of Runx3 sustains the production of IFN-γ in Th17 cells [15]. 

Environmental factors, such as diet or exposure to cigarette smoke, play a pivotal role in the immune responses to GI inflammation [1]. They affect primary epigenetic mechanisms, such as DNA methylation, and, thus, may modulate the molecular basis of cellular memory and transcriptional switching of immune gene expression [1,16]. The methylated form of the DNA base may restrain transcription factors from binding to DNA and activate transcriptional repressor proteins [1]. The methylation status of cytosine residues in promoter-associated CpG sites was previously identified as a hallmark of UC-related colorectal carcinogenesis [17]. Although the molecular basis of chronic inflammation is being explored, methylation-dependent mechanisms are still largely unidentified [14]. Thus, the complex interplay between genetic and epigenetic factors contributing to colitis onset and progression requires further study. Here, we determined the *RUNX3 P2* methylation profile and its association with pediatric UC activity. We analyzed the dependence of epigenetic changes on patients’ age, sex, nutritional status, level of acute-phase proteins, and exposure to pharmacotherapy, as well as the disease onset and activity and extent of colitis.

## 2. Materials and Methods

### 2.1. Patient Recruitment

Patient recruitment took place during scheduled visits and hospitalization at university clinics in Poznan, Warsaw, Bydgoszcz, Zabrze, and Katowice (April 2016–March 2019, Poland). Experienced gastroenterologists confirmed UC diagnoses. The extent of mucosal inflammation was assessed using endoscopic appearance. Identification of E1—ulcerative proctitis, E2—left-sided UC, E3—extensive UC, and E4—pancolitis followed the Paris classification. The Pediatric Ulcerative Colitis Activity Index was noted as: S0—never severe disease / S1—ever severe disease. Symptoms such as abdominal pain, rectal bleeding, stool consistency, stool frequency, nocturnal stools, and limitations in patient activity corresponded to the index cut-offs of remission (<10) and mild (10–34), moderate (35–64), and severe UC (65–85). All life-threatening conditions were excluded from the study.

### 2.2. Group Characteristics

This cross-sectional study was performed on blood samples obtained from 64 pediatric patients with UC. There were 33 (51.6%) female and 31 (48.4%) male children. The age ranged from 6 to 17 years, with a mean of 14 years in each group. Clinically, the extent of UC included pancolitis (48.4%, Paris E4), left-sided UC (18.8%, E2), ulcerative proctitis (17.2%, E1), and extensive UC (15.6%, E3). Individuals were divided into subgroups following the Paris endoscopic classification, the pediatric UC activity index (PUCAI), nutritional status, and C-reactive protein (CRP) and albumin levels at inclusion. The mean duration of disease among all patients was the longest for individuals in remission. The majority of patients received multiple-drug therapy. Sample characteristics are described in Table 1.

### 2.3. Genomic DNA Isolation

Briefly, samples were incubated in LT lysis solution and proteinase K for 20 min at 37 °C to lyse erythrocytes in whole blood. Total genomic DNA was isolated from the whole blood of 64 UC patients using Blood Mini microcolumns according to the manufacturer’s protocol (A&A Biotechnology, Gdansk, Poland). Subsequently, DNA elutions in low-salt Tris buffer (10 mM, pH 8.5) were stored at −80 °C until further investigation. We used the Thermo Scientific NanoDrop Lite spectrophotometer to determine dsDNA concentration and its purity in each sample. As the final reaction was optimized for 20 ng input, DNA concentration was standardized to 4 ng/µL before the experiment.

### 2.4. Primer Design

**Figure 1:**
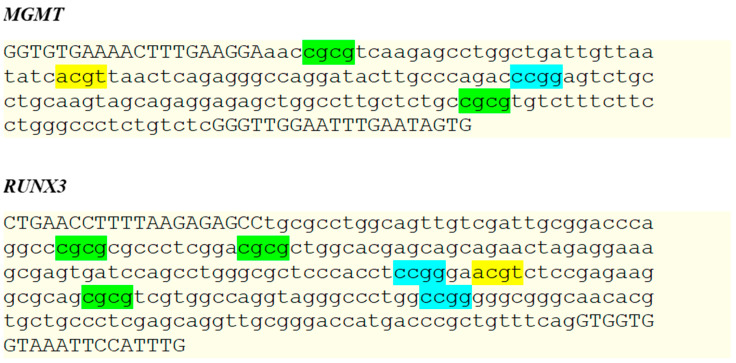
Expected products for *MGMT* and *RUNX3* primer sets. We designed *RUNX3* primers to obtain a product 150 to 350 bp long with at least 2–4 MSRE sites. A mix of restriction enzymes was used for each amplicon. Capital letters indicate primer sequences. Green, blue, and yellow highlight restriction sites specific to AccII, HpaII, and HpyCH4IV, respectively.

The *MGMT* (O-6-methylguanine-DNA methyltransferase) primer set, provided with a OneStep qMethyl kit (Zymo Research, Orange, CA, USA), was used as a reference to create primers for the *RUNX3* gene. We utilized the NCBI Primer-BLAST to design oligonucleotides. The FASTA-formatted *RUNX3* sequence was analyzed for CpGs number, optimal product length, expected methylation status of cytosines, and methylation-sensitive restriction enzyme (MSRE) recognition sites. Thus, our design aimed to bind within the targeted CpG island and contain at least two, but no more than six, MSRE sites in a product ranging from 150 to 350 bp long. Subsequently, in silico primer tests were obtained via the primer tool available at https://genome.ucsc.edu (accessed on 13 June 2022) to verify the applicability of selected oligonucleotides for our reaction conditions. *RUNX3* primers were synthesized and delivered by Genomed (Warsaw, Poland). We diluted primers in DNase/RNase-free water to obtain a final concentration of 10 µM. Primers used in this study and graphical representations of each methylation-specific recognition site within the qPCR products are shown in Table 2 and Figure 1, respectively.

### 2.5. MSRE-qPCR Conditions

The methylation level of the *RUNX3* promoter was determined using the OneStep qMethyl Kit from Zymo Research. Analyses were performed in duplicate for each experiment. The final reaction volume of 20 µL contained premix with SYTO 9 dye, 10 pmol/µL of each primer, and 5 µL of DNA template. Reactions were carried out in the presence (test reaction) or absence (reference reaction) of MSRE (AccII, HpaII, and HpyCH4IV), as per the manufacturer’s guideline. Cycling conditions were as follows: MSRE digestion (37 °C, 2 h), initial denaturation (95 °C, 10 min), 40 cycles of three-step amplification (denaturation: 95 °C, 30 s; annealing: 54 °C, 60 s; extension: 72 °C, 60 s), and final extension (72 °C, 7 min). In addition, amplified products were melted in a temperature gradient to a maximum of 95 °C.

### 2.6. Assessment of DNA Methylation

Obtained results were related to fully methylated and non-methylated standards of human DNA (OneStep qMethyl Kit, Zymo Research, USA). The samples were also normalized with three independent samples, which were measured in duplicate in each pool. The *RUNX3 P2* methylation level of each sample was assessed through a comparison of the real-time amplification plots for PCR products and standards with a known ratio of methylated and unmethylated templates. A cycle threshold value was based on 5-methylcytosine content at the specific restriction site. As restriction enzymes recognized and cut unmethylated nucleotides between 3′ hydroxyl and 5′ phosphate groups, templates without methylation obtained higher Ct differences. The methylation level for a region spanned by the *RUNX3* primers was established as a fold-change relative to Ct value differences for test and reference reactions, following a formula: methylation (%) = 100 x 2^−ΔCt^. Product characteristics and purity were determined using melting curves and peak analysis under the control of CFX Manager software. 

### 2.7. Statistical Analysis

We examined population characteristics using Statistica 13.1 software. Variables were measured on interval, ordinal, and nominal scales. Thus, descriptive statistics and verification of normal distribution were required. We set a statistically significant *p*-value as <0.05 for parametric and nonparametric analyses. Parametric tests, such as independent t-tests and one-way ANOVA, were mostly applied to methylation datasets. If variables were ordinal or nominal, we chose nonparametric tests. These analyses included the Mann–Whitney U test, the Kruskal–Wallis (K-W) test, the K-W analysis of variance, and the Chi^2^ test. Additionally, a forward stepwise linear regression was built to determine the relationship between *RUNX3 P2* methylation level and biological treatment. The following confounders were included: age, sex, and CRP.

### 2.8. Ethical Considerations

The study was conducted in accordance with the criteria set by the Declaration of Helsinki. Approval of the protocol was obtained from the Bioethical Committee at Poznan University of Medical Sciences (no. 960/15). All patients/guardians provided informed written consent for participation in the study, as regulated by the local law.

## 3. Results

**Figure 2:**
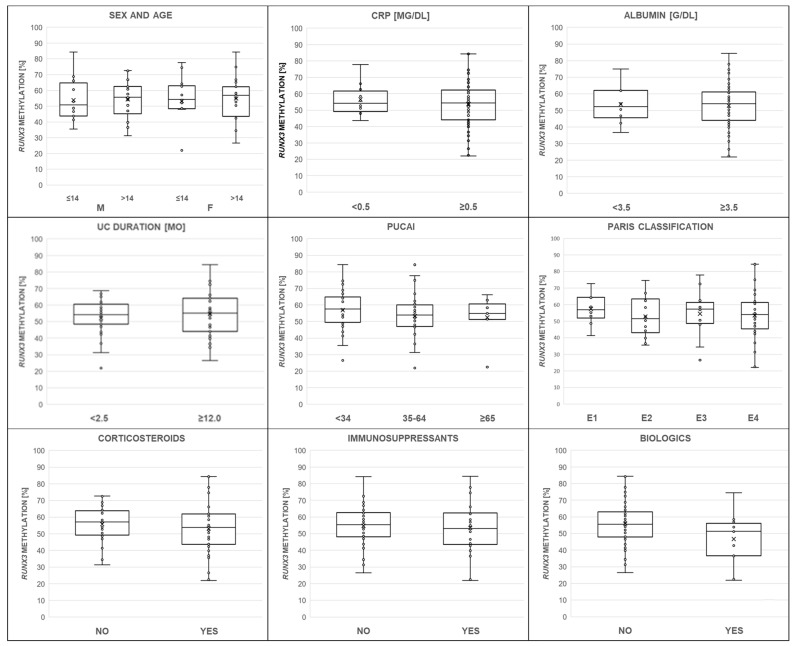
The relationship of the *RUNX3* methylation level (%) to clinical characteristics such as patients sex and age, C-reactive protein (CRP), serum albumin, disease duration, Pediatric Ulcerative Colitis Activity Index (PUCAI), colitis extent according to the Paris classification (E1–E4), and exposure to medications (corticosteroids, immunosuppressants, and biologics, respectively). The mean is indicated with an “x” mark. Additionally, the median is shown as a horizontal line within box plots. None of the obtained associations were considered significant (*p* > 0.05).

The distribution of *RUNX3 P2* methylation ranged from 22.0% to 84.4%, with the mean level at 54.1 ± 13.3%, in DNA samples from children with UC. Figure 2 shows *RUNX3 P2* methylation changes dependent on patients’ age, sex, level of acute-phase proteins, and exposure to pharmacotherapy, as well as disease onset and activity and the extent of colitis.

### 3.1. Demographic Factors

The studied population presented a balanced sex ratio and age profile. Girls and boys with UC had similar *RUNX3 P2* methylation levels (54.2% vs. 54.1%, *p* = 0.9783). Methylation of *RUNX3* remained similar across age groups. *RUNX3 P2* methylation did not differ depending on children’s age (over 14 years of age vs. the rest, 54.5% vs. 53.5%, *p* = 0.7743).

### 3.2. Nutritional Status

Body mass index (BMI) significantly increased with the children’s age (*p* < 0.05, r_P_ = 0.2944), as expected. The BMI Z-score associations did not correlate with *RUNX3 P2* methylation level (*p* > 0.05, r_S_ = 0.0133). The baseline nutritional status described by the weight-for-length/height Z-score was −0.44 ± 1.14.

### 3.3. Inflammatory Markers

**Figure 3:**
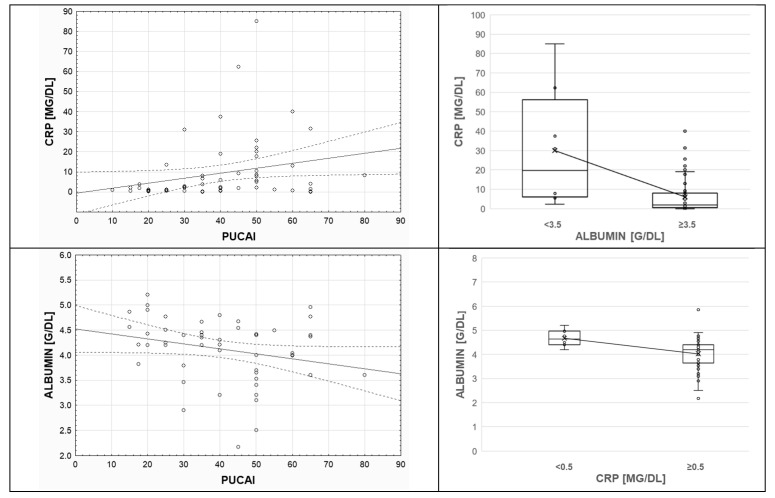
Inflammatory markers’ associations with Pediatric Ulcerative Colitis Activity Index (PUCAI) scores and pairwise associations. Colonic inflammation was associated with higher C-reactive protein (CRP) and lower albumin levels in children with UC. Serum albumin tended to decrease as the values of CRP increased (*p* < 0.05). The “x” mark indicates the mean value. The horizontal line within the box plot shows the median.

Among the 64 children, more than half (66.0%) had high CRP, considered as greater than the 1 mg/dL cutoff (normal CRP <0.5 mg/dL). This positive acute-phase marker significantly increased with inflammatory burden, as expressed in higher PUCAI scores (*p* < 0.05, r_S_ = 0.3273) and shown in Figure 3. Thus, baseline PUCAI groups were separated; defined as remission (<10), mild (10–34), moderate (35–64), and severe (≥65) disease activity; and compared. Subset analyses showed that CRP medians differed according to PUCAI subgroups (H = 10.5167, *p* = 0.0052, and Chi^2^ = 10.7048, *p* = 0.0047), but methylation of *RUNX3 P2* did not depend on CRP (56.47% vs. 53.67%, 0.5 mg/dL cutoff, *p* = 0.5534).

In the investigated group, albumin negatively correlated with CRP levels (*p* < 0.05, r_S_ = −0.5529) and PUCAI scores (*p* < 0.05, r_S_ = −0.2904). Figure 3 illustrates these trends. Pediatric population predominantly (75.0%) displayed normal serum albumin concentration (reference range: 3.5–5.2 g/dL). *RUNX3 P2* methylation did not differ in subjects with low and normal albumin (<3.5 vs. ≥3.5 g/dL, 53.68% vs. 52.83%, *p* = 0.8699).

### 3.4. Disease Characteristics

The PUCAI, which integrates information on symptoms such as abdominal pain, rectal bleeding, consistency of most stools, number of stools per day, nocturnal stools, and patient activity level, provided objective measures for disease activity. Using PUCAI scores, we assessed the potential for the use of *RUNX3 P2* methylation to discriminate between mild, moderate, and severe disease. The analyzed comparisons were considered statistically insignificant (57.5% vs. 53.9% vs. 54.6%, H = 1.3309, *p* = 0.5140). Moreover, the levels of *RUNX3 P2* methylation, expressed as medians, did not differ across E1–E4 colitis (sequentially: E1 56.9% vs. E2 51.5% vs. E3 57.2% vs. E4 54.0%, H = 0.9412, *p* = 0.8155, and Chi^2^ = 1.5077, *p* = 0.6805).

Patients were also separated into early- and late-onset groups, defined by the age ranges 0–7 and 8–18 years, to assess *RUNX3* methylation level depending on UC course. *RUNX3 P2* methylation did not correlate with patient age (*p* = 0.7031). Among all children, 48.4% received the diagnosis less than 2.5 months prior to recruitment and 42.2% over a year earlier. Six cases that were enrolled between these time points were excluded from this analysis. Individuals with a longer time from diagnosis developed only nominally higher DNA methylation within the *RUNX3* promoter sequence (52.1% vs. 54.9%, *p* = 0.4104). The correlation between gene methylation level and disease duration was also insignificant. 

### 3.5. Applied Therapeutics

Among all children with UC, 39 (60.9%) received corticosteroids, 27 (42.2%) immunosuppressants, and 12 (18.8%) biologics. Nine individuals received all of the major UC medication groups. Distribution of cortisone-like (Chi^2^ = 10.6136, *p* = 0.0050), immunosuppressive (Chi^2^ = 11.3723, *p* = 0.0034), and biologic (Chi^2^ = 9.4050, *p* = 0.0091) drugs increased with PUCAI scores. Children with corticosteroids did not have significantly increased or reduced *RUNX3 P2* methylation levels compared to individuals without treatment (53.2% vs. 55.7%, *p* = 0.4105, r_S_ = −0.1061). Such an effect was also not found for patients on immunosuppressants (53.5% vs. 54.6%, *p* = 0.6640, r_S_ = −0.0564), as shown in Table 3. The highest nominal, but not statistically significant, decrease in *RUNX3* promoter methylation level occurred in children receiving biological treatment (46.7% vs. 55.9%, *p* = 0.0569, r_S_ = −0.2423). In regression analysis, methylation of *RUNX3 P2* correlated with biological treatment independently of age, sex, and CRP (methylation beta = −0.2701, 95%CI −0.5187-(−0.0214); model R2 = 5.74%, *p* = 0.0337).

## 4. Discussion

Runt-related transcription factor 3 (*RUNX3*) reveals functional duality in the pathogenesis of inflammatory bowel disease [18]. It is predominantly expressed in hematopoietic lineages and transcribed from two promoters: P1 that is distal and CpG-poor, and P2 which is located proximally within a conserved CpG [11,19,20]. Methylation of promoter CpG sites may lead to *RUNX3* transcriptional silencing. Subsequently, dysregulation of RUNX3-associated pathways disrupts the immune cells’ development, responses, or inflammatory cytokines synthesis [10,18]. In this work, we described changes in local DNA methylation at *RUNX3 P2* in the whole blood of 64 children with UC. The investigated site was highly methylated. However, no strong associations were found between the methylation level and clinical characteristics of UC. 

### 4.1. RUNX3 and Inflammation

The observation of the methylated *RUNX3 P2* sequence in UC was not entirely unexpected, as *RUNX3* is located within an IBD susceptibility locus [8,21]. *RUNX3* has been implicated in neutrophil/T lymphocyte regulation; its functional loss may increase the risk for UC [8] and facilitate UC-associated tumor growth [21,22]. This was demonstrated in both animal models and human cohorts. Brenner et al. showed that leukocytes that have lost their autonomous function due to *Runx3* knockout contribute to the occurrence of early-onset colitis and gastric lesions in mice [12]. Knowing that epigenetic changes may affect gene expression, a strong inverse correlation between *RUNX3* methylation and expression was demonstrated in a study by Tserel et al. [23]. Due to *RUNX3* association with T-cell differentiation, this suggests a link between functional impairment of immunity cells and methylation pattern remodeling in chronic inflammation characteristic of UC.

Chronic, low-grade inflammation also constitutes a typical feature of aging [23]. Pro-inflammatory response is more prominent in females [24]. It favors the accumulation of epigenetic changes, including hypermethylation of CpG islands, which might explain impaired immunocompetence [23]. Observations in this field have come almost exclusively from studies of adults with IBD. Several studies of blood noted sex- and age-specific effects on DNA methylation profiles in CD8+ T lymphocytes [23,25]. Tserel et al. [23] highlighted increasing methylome variation with *RUNX3* hypermethylation in ageing CD8+ T cells. Expression of *RUNX3* directs T-cell differentiation towards mature CD8+ lymphocytes. In keeping with these reports, Gasparetto et al. [25] distinguished IBD phenotypes with childhood- and adult-onset. Although the naive T-cell pool underwent reduction with age, hallmarks of a CD8+ T-cell expression were not found in pediatric patients. In this context, we correlated methylation data with children’s demographics, such as sex ratio and age structure. Our current study provides evidence that the methylation level of *RUNX3* alone is insufficient to discriminate between differences in UC pediatric cohorts.

Intestinal inflammation contributes to chronic weight loss due to nutrient malabsorption. Even though malnutrition remains one of the major concerns in IBD, current studies indicate that the nutritional status of UC patients changes towards excessive body weight comparably to trends in the general population [26,27]. The risk of obesity is 3.5 times higher in UC patients compared to those with Crohn’s disease (CD) [27], and approximately one in three children with UC are overweight [26]. As DNA methylation reveals age specificity, it might also be affected by body mass growth. However, epigenomic studies showed a lack of a causal relationship between DNA methylation status and body mass index (BMI). Only a tendency towards increasing variability in methylation patterns was found, overlapping across childhood and adolescence [28,29]. These findings are compatible with our *RUNX3 P2* observations, which showed an increase in gene methylation levels in children with higher BMI Z-scores.

Adiposity is known to drive cytokine-mediated inflammatory signals. Irrespective of BMI, the available literature indicates levels of serum C-reactive protein and albumin as reliable predictors of inflammation and, therefore, UC severity [30,31]. Ventham et al. [32] demonstrated that outcome prediction in IBD subclasses was related to the CRP/albumin ratio. This is in line with our observations of increasing levels of CRP and decreasing albumin with inflammatory burden, as expressed in high PUCAI scores. While CRP seems to reflect active mucosal inflammation in the colon [30], serum albumin positively correlates with mucosal healing [33]. Thus, hypoalbuminemia enables clinical course prediction at diagnosis [34]. The implications of *RUNX3* in these findings remain unclear. However, studies of inflammatory diseases, such as ankylosing spondylitis, show allele-specific effects on *RUNX3.* Nevertheless, gene expression did not correlate with CRP, which was used to describe disease activity [35]. Although we did not perform expression analysis, our work suggests that *RUNX3 P2* methylation does not relate to serum CRP or albumin in pediatric UC.

### 4.2. UC in Children and Observations Regarding RUNX3 in This Study

Differential diagnosis of UC vs. CD in new-onset pediatric IBD remains challenging despite the use of complex diagnostics that include expert endoscopy and grading. These studies involve general anesthesia and cannot be used to monitor disease activity at the frequency necessary for proactive inflammation detection and management. Thus, the employment of noninvasive inflammatory biomarkers seems a practical way to determine disease activity. Most elderly patients develop proctitis or left-sided colitis [36]. A few pediatric studies have suggested the predominance of pancolitis with a more aggressive course in early-onset UC [36,37]. By the age of seven, children are more frequently reported to have pancolitis with long diagnostic delays and extraintestinal manifestations [37]. In accordance with the literature, we noted a high prevalence of pancolitis in this nationwide pediatric population. Reactive oxygen species may accumulate during longstanding UC, triggering DNA hypermethylation [21]. Howell et al. [38] determined numerous IBD-specific changes in DNA methylation levels in children, but no data on the *RUNX* family were mentioned. Our current work showed that methylation of *RUNX3 P2* is not associated with the clinical characteristics of pediatric UC. Colitis extent and symptom severity were similar between analyzed subgroups at diagnosis/inclusion. 

The pediatric onset of colitis is often associated with a more extensive and aggressive disease compared with that in adulthood. This may result in frequent early use of steroids, immunosuppressants, and, sometimes, biologics. Both DNA methylation and applied therapeutics might modify the effectiveness of specific treatments. Thus, aberrant methylation within *RUNX3 P2*, depicted as either hypo- or (more probably) hypermethylation, may disrupt genome-wide functional patterns responsible for immune responses. Although our cross-sectional study design limited prediction of cause–effect relations, we did not observe considerable differences in *RUNX3* methylation status based on medications received. Samples obtained from children on biologics appeared to have the lowest DNA methylation at the investigated gene locus. Our observations corresponded with previous studies on the requirement for escalation of medications, especially biologics inclusion, within the first 18 months after the diagnosis [37,38]. Patients with early onset of colitis developed neither steroid dependency nor resistance [36,37].

### 4.3. Limitations

There were certain limitations to this study. The studied population was heterogeneous, which reduced the subgroup sizes available for *t*-test (or equivalent) analyses but also broadened the possibilities of correlation analysis. A cross-sectional study design allowed us to examine the presence/absence of outcomes at a specific point in time but without follow-up. The accuracy of the employed assays may have been limited, but we applied them in a number of samples to better understand the potential for personalized medicine in IBD. Although our analysis lacked a healthy control group and standardization per leukocyte subtypes, the obtained data provide insight into the epigenetics of *RUNX3* in a substantial pediatric cohort. It must be mentioned that results from children should not be generalized to adults, in whom methylation may be affected by longer-term environmental exposures. Notable geographical variation in UC has also been observed. Therefore, results from one region should not be directly generalized to the global population, as UC epigenetics may have population-specific characteristics.

## 5. Conclusions

The exact cause of prolonged inflammation of the colon in children remains unknown. We took the opportunity to investigate the potential implication of *RUNX3 P2* in UC pathophysiology. Knowing that expression of *RUNX3* may orchestrate immune-cell plasticity and differentiation, it would be useful to understand whether epigenetic hallmarks, such as DNA hypermethylation, play a pivotal role in triggering and/or maintaining autoimmunity in children. We believe that the results obtained from the Polish nationwide pediatric cohort may inspire readers to develop new ideas on the relationship of *RUNX3* with both UC characteristics and the environment–microbiota–immunity axis.

In summary, methylation of *RUNX3* promoter 2 in whole-blood DNA does not seem to be associated with the clinical characteristics of UC in children. Although *RUNX3 P2* methylation levels did not differ depending on pharmacotherapy, the hypothesis that biological treatment reduces *RUNX3 P2* methylation and increases RUNX3 signaling requires further study.

## Figures and Tables

**Table 1:** Sample characteristics.

Characteristic	Sample	
**Total no. of patients [*n*]**	64	
Average age [yr]	14.5 ± 2.8	
Average BMI [kg/m^2^]	18.0 ± 3.2	
Average BMI Z-score	−0.44 ± 1.14	
**Group**	**Female**	**Male**
No. of patients [*n* (%)]	33 (51.6%)	31 (48.4%)
Average age [yr]	14.6 ± 2.6	14.4 ± 3.1
Average weight [kg]	43.7 ± 18.1	44.0 ± 17.8
BMI [kg/m^2^]	18.0 ± 3.4	18.0 ± 2.9
Average duration of the disease [yr]	1.7 ± 2.3	2.6 ± 3.3
<2.5 mo. since UC diagnosis [*n* (%)]	15 (45.5%)	16 (51.6%)
≥12 mo. since UC diagnosis [*n* (%)]	14 (42.4%)	13 (41.9%)
Baseline PUCAI [point]	39.8 ± 17.3	38.7 ± 16.4
<10 [*n* (%)]	0 (0.0%)	0 (0.0%)
10–34 [*n* (%)]	12 (36.4%)	10 (32.3%)
35–64 [*n* (%)]	16 (48.5%)	14 (45.2%)
≥65 [*n* (%)]	4 (12.1%)	3 (9.7%)
Baseline Paris Classification	E3	E4
E1 [*n* (%)]	6 (18.2%)	2 (6.5%)
E2 [*n* (%)]	6 (18.2%)	6 (19.4%)
E3 [*n* (%)]	6 (18.2%)	4 (12.9%)
E4 [*n* (%)]	14 (42.4%)	17 (54.8%)
S0 [*n* (%)]	23 (69.7%)	27 (87.1%)
S1 [*n* (%)]	9 (27.3%)	3 (9.7%)
Baseline CRP [mg/dL]	6.8 ± 10.7	10.9 ± 19.2
Baseline albumin [g/dL]	4.2 ± 0.6	4.0 ± 0.7
Concomitant pharmacotherapy [*n* (%)]	21 (63.6%)	20 (64.5%)
Systemic corticosteroids [*n* (%)]	19 (90.5%)	20 (100.0%)
Immunosuppressants [*n* (%)]	15 (71.4%)	12 (60.0%)
Biologics [*n* (%)]	8 (38.1%)	4 (20.0%)

Means ± SD are shown. The analyzed features did not differ significantly depending on gender (*p* > 0.05). Among the 64 patients recruited, 6 individuals were excluded from the part of the statistical analyses referring to disease duration, and 3 were excluded due to disease extent, and 5 due to UC activity. Baseline PUCAI defines disease severity as: remission (<10), mild (10–34), moderate (35–64), severe (≥65). The baseline Paris Classification presents the median. Disease extent/severity is defined as follows: E1 ulcerative proctitis, E2 left-sided colitis, E3 extensive colitis, E4 pancolitis, S0 never severe, S1 ever severe. Concomitant medication includes corticosteroids (methylprednisolone, prednisone, hydrocortisone), immunosuppressants (azathioprine, methotrexate, mercaptopurine, cyclophosphamide, mycophenolic acid, tacrolimus), and biologics (infliximab, adalimumab).

**Table 2:** Primer sequences.

Gene	Primer Sequence 5′ → 3′		Ta [°C]	MSRE Sites [*n*]/Amplicon Size [bp]	Reference
*MGMT*	F	GGTGTGAAAACTTTGAAGGA	54	4/186	Zymo Research design
	R	CACTATTCAAATTCCAACCC			
*RUNX3*	F	CTGAACCTTTTAAGAGAGCC	54	6/264	Designed using NCBI Primer-BLAST
	R	CAAATGGAATTTACCACCAC			

F, forward primer; R, reverse primer; Ta, annealing temperature.

**Table 3:** Methylation of *RUNX3* vs. applied therapeutics.

Medication	Received	*RUNX3 P2* Methylation Level [%]			*p*-Value
		N	Min–Max	Mean ± SD	
Corticosteroids	no	24	31.3–72.7	55.7 ± 10.7	0.4105
	yes	39	22.0–84.4	53.2 ± 15.0	
Immunosuppressants	no	36	26.5–84.3	54.6 ± 12.1	0.6640
	yes	27	22.0–84.4	53.5 ± 15.2	
Biologics	no	51	26.5–84.4	55.9 ± 12.5	0.0569
	yes	12	22.0–74.5	46.7 ± 15.3	

Analyzed courses of medication included corticosteroids (methylprednisolone, prednisone, hydrocortisone), immunosuppressants (azathioprine, methotrexate, mercaptopurine, cyclophosphamide, mycophenolic acid, tacrolimus), and biologics (infliximab, adalimumab). One sample was excluded from the statistical analysis.

## Data Availability

The data that support the findings of this study are available from the corresponding author upon reasonable request.
